# Combining Exergy and Pinch Analysis for the Operating Mode Optimization of a Steam Turbine Cogeneration Plant in Wonji-Shoa, Ethiopia

**DOI:** 10.3390/e26060453

**Published:** 2024-05-27

**Authors:** Shumet Sendek Sharew, Alessandro Di Pretoro, Abubeker Yimam, Stéphane Negny, Ludovic Montastruc

**Affiliations:** 1 School of Chemical and Bioengineering, Addis Ababa Institute of Technology (AAiT), Addis Ababa University, Addis Ababa P.O. Box 385, Ethiopia; shumet.sendek@aait.edu.et (S.S.S.); abubeker.yimam@aau.edu.et (A.Y.); 2 Laboratoire de Génie Chimique, Université de Toulouse, CNRS/INP/UPS, Allée E. Monso 4, 31432 Toulouse, France; stephane.negny@ensiacet.fr (S.N.); ludovic.montastruc@ensiacet.fr (L.M.)

**Keywords:** pinch analysis, exergy, cogeneration plant, operating scenarios, ProSimPlus^®^

## Abstract

In this research, the simulation of an existing 31.5 MW steam power plant, providing both electricity for the national grid and hot utility for the related sugar factory, was performed by means of ProSimPlus^®^ v. 3.7.6. The purpose of this study is to analyze the steam turbine operating parameters by means of the exergy concept with a pinch-based technique in order to assess the overall energy performance and losses that occur in the power plant. The combined pinch and exergy analysis (CPEA) initially focuses on the depiction of the hot and cold composite curves (HCCCs) of the steam cycle to evaluate the energy and exergy requirements. Based on the minimal approach temperature difference (∆*T_lm_*) required for effective heat transfer, the exergy loss that raises the heat demand (heat duty) for power generation can be quantitatively assessed. The exergy composite curves focus on the potential for fuel saving throughout the cycle with respect to three possible operating modes and evaluates opportunities for heat pumping in the process. Well-established tools, such as balanced exergy composite curves, are used to visualize exergy losses in each process unit and utility heat exchangers. The outcome of the combined exergy–pinch analysis reveals that energy savings of up to 83.44 MW may be realized by lowering exergy destruction in the cogeneration plant according to the operating scenario.

## 1. Introduction

During the last decades, the efficient use of energy has become one of the aspects of major interest in a wide range of engineering fields. This increasing attention is due to both the particular concerns toward the environmental impact of industrial processes and to the relevant potential in terms of energy consumption reduction, and thus operating costs. In the process engineering domain, the best-established solutions for the mitigation of heating and cooling utilities are represented by process intensification and energy integration. The former involves the use of intensified equipment, able to perform multiple operations inside the same unit [1], while the latter consists of the rearrangement of the utility network with the purpose of using the process streams to be cooled as hot duty for the process streams to be heated and vice versa [2].

Regarding the second method, to increase the efficiency of energy usage in the heat exchanger network (HEN), a systematic approach called pinch analysis is conventionally employed. However, as Zhao et al. (2022) [3] point out, the primary drawback of pinch analysis is that it can only address processes involving heat transfer while it cannot address processes from the perspective of pressure and composition variations. All the stream parameters such as pressure, temperature, and composition that could potentially mitigate the drawback of pinch analysis are instead included in the so-called exergy analysis. The combination of exergy and pinch analysis applied to industrial processes is essential to determine which operations cause the greatest number of exergy casualties in heating and power facilities. Exergy is defined as the maximum amount of work that may be obtained from a certain thermal system as it moves towards a specific ultimate state when it is in balance with its environment. As a result of internal or external irreversibilities, exergy is not conserved as energy, and it is destroyed inside the system. To solve the internal or external irreversibilities in a process system other than energetic performance, which is based on the first law of thermodynamics, and exergetic performance, which is based on the second law, pinch technology, which is based on both, can be exploited. This work seeks to provide a novel combined pinch and exergetic-based systematic method for evaluating and enhancing current industrial processes from an energy perspective.

In order to integrate these two fragmented methodologies, a new technique called combined pinch and exergy analysis (CPEA) is applied to a cogeneration plant case study in this research. In particular, the plant simulation is based on the actual operating data of the Wonji-Shoa (Ethiopia) combined heat and power plant that provides the steam utility for the related sugar factory and, in addition, generates power for the national electricity grid. For a regular operating flow, the typical steam turbine is designed to ensure the turbo-alternator inlet steam flow of up to 164 t/h at 64 bar pressure and 505 °C temperature with uncontrolled extraction at 9 bar. At the desuperheater’s output, the steam flowrate is equal to 20 t/h with regulated extraction at a pressure of 2.6 bar, resulting in a maximum steam flow of 117 t/h when the unit is operating at full capacity. Once the simulation to obtain all the other operating parameters is performed by means of ProSimPlus^®^, the CPEA can be then carried out. For the illustration of process streams on a temperature versus enthalpy (T–H) diagram, Linnhoff et al. (1982) [4] developed the composite curves (CCs), which are a common pinch analysis graphical tool. The hot or cold CCs are built from a combination of hot or cold streams that operate within a predetermined temperature range. To achieve the greatest amount of heat recovery potential and the least amount of hot and cold utility needs, the composite hot and cold streams can be horizontally shifted to approach one another along the enthalpy (∆*H*) axis, until they are nipped off. The visual features of the cold and hot composite curves (CCs) and the grand composite curve (GCC) make it easier to find chances for heat integration, and they are highly helpful for gaining an in-depth knowledge of the issue [5]. The minimal energy objective for the operation may be then determined with the constraint of heat transfer going from higher to lower temperatures by keeping a minimum temperature difference as the driving force. Furthermore, the minimal permitted temperature differential Δ*T_lm_* also represents a financial indicator of a nearly ideal trade-off between the cost of the initial investment (heat exchanger unit(s)) and the cost of running the process (energy) [6].

In this research, the exergy–pinch analysis method addresses the three possible functioning modes of the Wonji-Shoa CHP plant, mostly focusing on the heat integration part for the enhancement of the processing systems’ energy efficiency. As better discussed in the dedicated section, each operation mode refers to a combination of the grid connection and sugar factory operating state (ON/ON, ON/OFF, OFF/ON). The variation in the electrical power provided by the plant for each scenario is managed by the regulation of the steam flowrate of up to 31.5 MW of useful power. The application of the exergy–pinch analysis could then highlight significant potential in terms of heat recovery when the process splits into a heat surplus zone below the pinch and a heat deficit region above the pinch. The main added value of combining pinch and exergy analysis with respect to standalone approaches, which are conventionally proposed, is represented by the possibility of simultaneously addressing both equipment technology and operation management improvements in the same analysis. Furthermore, since both the methodologies are suitable for energy and process systems that either already exist or need to be designed, when combining the two there is no need for methodological adaptations.

Better details about the specific application of these methodologies on the selected case study, along with the operating parameters resulting from the process simulation, are then provided in the following section.

## 2. The Wonji-Shoa Case Study

**Figure 1:**
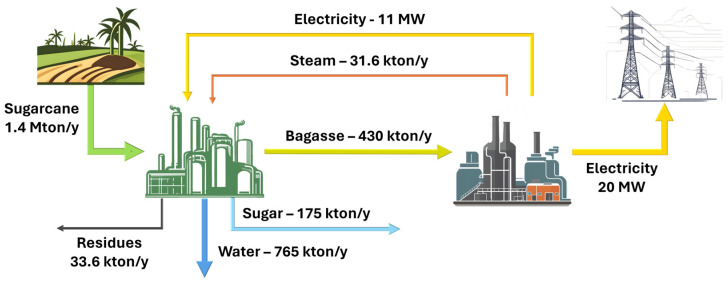
Simplified flow diagram of the Wonji-Shoa facility.

As mentioned in the introduction, the selected system for this research is an existing 31.5 MW cogeneration plant located in Ethiopia whose purpose is both to provide steam to the related sugar factory and to produce electricity for the national grid. The main advantages of this choice are the availability of the actual operating parameters [7] and the fact that the improvements obtained from the CPEA study could be effectively implemented in the real system. For a detailed analysis and discussion regarding the process side optimization, the reader could refer to the previous work of the authors [8] where the aspects concerning the plant operation are thoroughly presented. A general overview of the system layout in terms of material and energy fluxes is nevertheless provided in Figure 1 to outline the capacity and utility distribution of the entire plant.

The present study focuses on the potential energy savings of the CHP section (grey block) and on the identification of possible operational upgrades to enhance energy efficiency as well as to further increase profitability. The Wonji-Shoa factory cogeneration plant was conceived to provide a 9 bar stream thermal duty at the desuperheater, 3 bar steam at the extraction, and for the supply of the electricity surplus to the grid for the maximization of the facility income. According to the required electricity demand and process operating conditions, the plant switches from one functioning mode to another throughout its yearly operation. In order to have a clear overview of the final results of this study, a brief description of the system units, simulated by means of ProSimPlus^®^, and the explanation of the three possible functioning modes are presented in this section.

**Figure 2:**
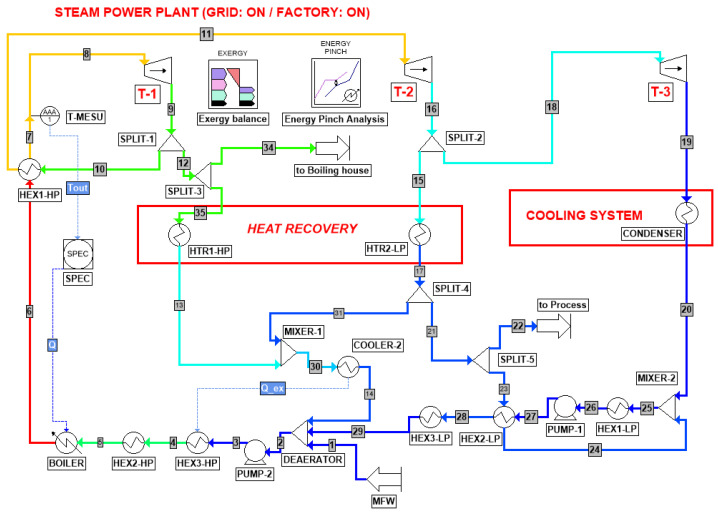
Steam turbine cogeneration plant for Scenario I (Grid: ON and Factory: ON).

The first operating mode, named Scenario I, refers to both power plant and sugar factory operation (Grid: ON/Factory: ON), and the corresponding system layout is reported in Figure 2. This is the default mode, and the turbo-generator capacity at full load corresponds to 31.5 MW. After the two steam extractions at 9 and 3 bar in correspondence of the turbines T-1 and T-2, the remaining steam is condensed and recycled with make-up water to reintegrate the water losses equal to 22% of the total flowrate. Once left the deaerator unit, whose purpose is to remove the dissolved gases, water feeds the boiler units whose heat is provided by the direct combustion of the bagasse by-product recovered from the sugar factory. The Scenario I functioning mode can be considered as the reference case since the other two result from layout modifications with respect to it.

**Figure 3:**
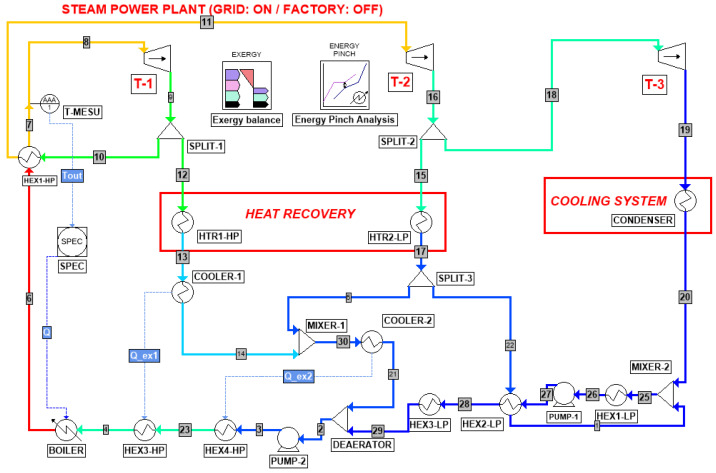
Steam turbine cogeneration plant for Scenario II (Grid: ‘ON’ and Factory: OFF).

Figure 3 shows the equivalent simulation layout for Scenario II (Grid: ON/Factory: OFF). Since the sugar factory is not operating, the absence of the stream splitting toward the process can be noticed. When running this mode, the cogeneration plant produces 130 ton/h of stream, with a reduced electricity production, and a single heat recovery solution was already implemented. Since there is no steam withdrawal, the make-up water valve can be closed, and the related mixing units are not active.

**Figure 4:**
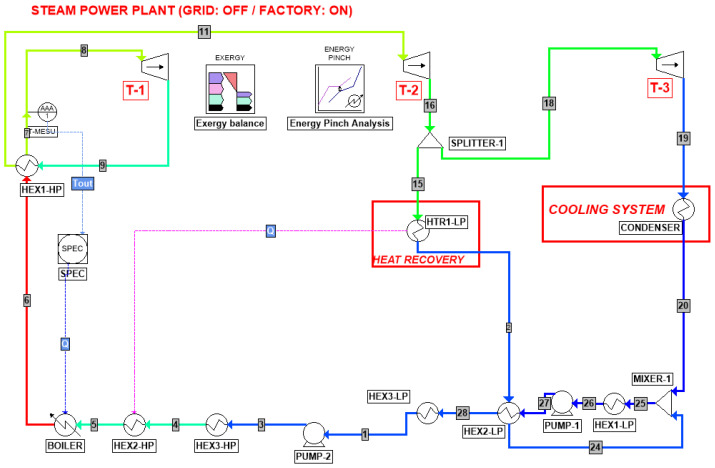
Steam turbine cogeneration plant for Scenario III (Grid: ‘OFF’ and Factory: ON).

Finally, in case the grid connection is down, Scenario III (Grid: OFF/Factory: ON) is represented by the process diagram in Figure 4. Since the sugar plant is the primary energy receiver, the turbine load can be decreased, and the boiler production is approximately equal to 59 ton/h of steam. As a consequence of the lower need for electricity production, there is no high-pressure extraction stream.

Based on this process layout description, the exergy and pinch analysis methodologies are therefore presented in the next sections by referring to the unit and stream names provided in Figure 2, Figure 3 and Figure 4.

## 3. Methodologies

Based on the process flow diagrams and the operating parameters available in the Wonji-Shoa factory manual [7], presented in the previous section, the research started with the implementation of the process simulation by means of ProSimPlus^®^. The reason lying behind the choice of this software is the availability of dedicated built-in tools for both pinch analysis and exergy balance, as can be pointed out in Figure 2, Figure 3 and Figure 4. However, in order to obtain the desired outcome from these two modules, they need to be properly set up according to the analysis constraints and the operating conditions. Therefore, methodological details concerning exergy analysis, pinch analysis, and CPEA are discussed in the respective following sections in order to facilitate the physical interpretation of the obtained results and to enable the correlation of the outcome with the preliminary hypotheses.

### 3.1. Exergy Analysis

In this study, the exergy analysis tool is used to detect the units in the system that exhibit higher irreversibility, i.e., exergy destruction. Exergy is defined as the highest potential useful work achievable from an energy carrier under the circumstances imposed by an environment at a certain pressure *P*_0_, temperature *T*_0_, and amount of chemical elements. The goal of the exergy analysis is to locate, source, and quantify actual thermodynamic inefficiencies in process plants, such as power plants (Chao and Yan, 2006) [9]. The advantage of using exergy for the evaluation of cogeneration system performance is based on the fact that instead of treating heat and electricity equally, as is the case with more traditional energy approaches, exergy methods enable the “value” of cogeneration products to be evaluated on an equivalent basis [10].

In this study, condensation-extraction steam turbines can exploit the entire amount of produced bagasse to increase the amount of excess power generated by the cogeneration system which is sold to the national grid [11]. In particular, in this simulation, the energy provided by the bagasse combustion is represented by the boiler’s heat duty term. The energy efficiency of a cogeneration facility producing both electricity and heat may be then defined as the ratio of useful energy output to the energy input given by Equation (1):

(1) $$\eta_{cogen}=\frac{{\dot{W}}_{net}+{\dot{Q}}_{heat}}{{\dot{E}}_{in}}$$

where ${\dot{W}}_{\mathrm{net}}$ is the net power output, ${\dot{Q}}_{heat}$ is the heating rate supplied by the plant, and ${\dot{E}}_{\mathrm{in}}$ is the rate of the total energy input to the plant.

Regarding the general form of the implementation of the exergy balance used by the ProSimPlus^®^ built-in module, it can be described by the following equation:

(2) $$\frac{dB_{cv}}{dt}=\sum_{j}\left(1-\frac{T_{0}}{T_{i}}\right)\cdot {\dot{Q}}_{j}-\left({\dot{W}}_{cv}-P_{0}\cdot \frac{dV_{cv}}{dt}\;\right)+\sum_{i}{\dot{m}}_{i}\cdot B_{i}-\sum_{0}{\dot{m}}_{0}\cdot B_{0}-\dot{I}$$

The specific physical exergy of a stream (*b_ph_*) is calculated by Equation (3):

(3) $$B_{ph}=\dot{m}\cdot [\left(h-h_{0}\right)-T_{0}\cdot (S-S_{0})]+B_{ch}$$

where $\dot{m}$ is the stream flowrate, *h* and *S* are, respectively, the specific enthalpy and entropy, and *B_ch_* is the term related to chemical exergy.

The specific physical exergy variation caused by temperature change is given by Equation (4):

(4) $$B^{T}=C_{p}\cdot \left(\left(T-T_{0}\;\right)-T_{0}\ln\frac{T}{T_{0}}\right)$$

while the specific physical exergy due to pressure change is given by Equation (5):

(5) $$B^{P}=R\cdot T\cdot \ln\frac{P}{P_{0}}$$

Finally, the specific chemical exergy, which can be calculated using conventional chemical exergy tables [12] in relation to environmental specifications, is given by

(6) $$B_{ch}=\sum_{i=1}^{k}X_{k}\cdot B_{k}^{CH}+{R\cdot T}_{0}\sum_{i=1}^{k}X_{k}\cdot \ln X_{k}$$

where *X_k_* and *R* are the molar fraction of the component *k*-th and the universal gas constant. In all equations, the subscript “0” indicates the reference conditions of the analysis that were set equal to the ambient temperature and pressure, i.e., 25 °C and 101.3 kPa, respectively.

The exergy destruction rate *İ* of a steady-state system is obtained from the equation

(7) $$\dot{I}=\sum_{j}B_{qj}\cdot {\dot{Q}}_{j}-\left({\dot{W}}_{cv}\right)+\sum_{i}B_{i}-\sum_{0}B_{out}$$

where *B_in_*, *B_out_*, and *B_D_* indicate the input exergy, output exergy, and exergy destruction of each unit, respectively. For the plantwide assessment of the exergy destruction, the unit-wise exergy destruction must be computed first. The bleed heat exchangers, condenser, expander, boiler, and auxiliary units lose some of the exergy from the fuel, while the remaining part is used to generate electricity with turbines. Finally, some exergy is wasted while turbines and pumps are working, according to their efficiency. The general energy and exergy efficiency is then equal to

(8) $$\eta_{I}=\frac{B^{out}}{B_{in}}=1-\frac{\dot{I}}{B_{in}}$$

According to Figure 2, Figure 3 and Figure 4, the exergy loss and the exergy efficiency for each of the steam turbine cogeneration cycle components may be computed as follows [13]:

(9) $$B_{\dot{I},turbine}=\sum {\left(\dot{m}\cdot b\right)}_{in}-\sum {\left(\dot{m}\cdot b\right)}_{out}-{\dot{W}}_{out}$$

(10) $$\eta_{Turbine}=\frac{{\dot{W}}_{out}}{\sum {\left(\dot{m}\cdot b\right)}_{in}-\sum {\left(\dot{m}\cdot b\right)}_{out}}$$

where ${\dot{W}}_{\mathrm{out}}$ is the actual generated expansion work.

The pump’s exergy loss and the related efficiency may be obtained as follows [14]:

(11) $$B_{\dot{I},pump}=B_{D,pump}=\sum {\left(\dot{m}\cdot b\right)}_{in}-\sum {\left(\dot{m\cdot }b\right)}_{out}+{\dot{W}}_{in}$$

(12) $$\eta_{pump}=\frac{\sum {\left(\dot{m}\cdot B\right)}_{out}-\sum {\left(\dot{m}\cdot B\right)}_{in}}{{\dot{W}}_{in}}$$

where ${\dot{W}}_{\mathrm{in}}$ is the actual consumed power.

As concerns the heat exchangers, the exergy balance is stated as

(13) $$B_{\dot{I},HE}=B_{in}-B_{out}=\sum {\left(\dot{m}\cdot B\right)}_{in}-\sum {\left(\dot{m}\cdot B\right)}_{out}$$

while the exergetic efficiency (*η_HEX_*) is given by the ratio of the rise in the cold fluid’s exergy to the reduction in the hot fluid’s exergy:

(14) $$\eta_{HEX}=\frac{\sum {\left[\dot{m}\cdot B_{out}-\dot{m}\cdot B_{in}\right]}_{Cold}}{\sum {\left[\dot{m}\cdot B_{out}-\dot{m}{\cdot B}_{in}\right]}_{Hot}}$$

Finally, the overall exergy loss in the cycle is given by the sum of all exergy losses in each involved unit operation, and the overall exergetic efficiency of the cycle can be finally calculated as

(15) $$\eta_{cycle}=\frac{{\dot{W}}_{net}}{B_{fuel=heat\;duty}}$$

where ${\dot{W}}_{\mathrm{net}}$ is the difference between ${\dot{W}}_{out}$ and ${\dot{W}}_{in}$:

(16) $${\dot{W}}_{net}={\dot{W}}_{out}-{\dot{W}}_{in}$$

### 3.2. Pinch Analysis

Pinch analysis targets energy-saving strategies by means of modifications in the heat exchanger network design based on heat balances and operating temperatures. In industrial settings, the calculation of the lowest heating and cooling requirements usually reveals considerable energy savings. Process and energy integration, particularly pinch technology, is a very effective analytical tool for the selection of technological solutions aiming at increasing efficiency and optimizing production. When integrating energy conversion technologies, one must take into account the combined production of heat and power and the integration of steam networks, heat pumps, and refrigeration systems, as well as how the minimum energy requirement will be met by converting energy resources into process-useful energy. Thus, the exergy concept is integrated with pinch analysis in the context of process integration analysis to reduce the energy requirement of the process [14], optimize energy conversion system integration, and introduce polygeneration.

The fundamental concept behind pinch is the possibility to independently depict process heating and cooling requirements by using composite curve (CC) diagrams [6]. CCs are graphical representations of temperature–enthalpy profiles for the hot (HCC) and cold (CCC) streams, representing the process heat availability and demand, respectively. When evaluating the energy efficiency of a process, pinch-based methodologies identify potential energy recovery via heat transfer and determine the process’s minimum energy requirement (MER). The heat exergy (*B_q_*) provided by a stream which delivers a heat load (Q) from *T_in_* to *T_out_* is estimated for each linear segment in an enthalpy–temperature curve computed by Equation (17):

(17) $$B_{q}=\dot{Q}\cdot \left(1-\frac{T_{0}}{T_{lm}}\right)$$

where *T_lm_* is the logarithmic mean temperature, and *T*_0_ is the ambient temperature.

The heat provided by the HCC is directly reported in the T–H diagram while the delivered exergy corresponds to the area between the composite curve and the enthalpy axis by replacing the temperature axis with the Carnot factor (1 − *T*_0_/*T*). The energy targets established by the composite curve (CC) and grand composite curve (GCC) in pinch analysis are exclusively expressed in terms of heat loads. However, in order to deal with systems including heat and power, the principles of both the CC and the GCC need to be expanded. A dedicated discussion about the shape and the interpretation of these graphical tools for the cogeneration plant case study are provided in Section 4. In addition, the GCC also depicts the difference between the available heat and the required amount.

Once the pinch has been discovered, the process may be divided into two distinct systems: one below the pinch and one above the pinch, as presented in Section 4 for this specific case study. In particular, the system behaves as a heat sink above the pinch and as a heat source below the pinch. Hence, in order to meet the process minimal energy targets, heat must not pass through the pinch, and there should not be external cooling and heating above and below the pinch, respectively. Thus, in case of insufficient heat in the hot streams above the pinch or insufficient cooling of the cold streams below the pinch, external utilities are required. According to Chen et al. (2016) [15], the overall goal of targeting multiple utilities is to maximize the usage of lower-cost utility levels while minimizing the use of higher-cost utility levels.

### 3.3. Combined Exergy and Pinch Analysis

In this study, a typical combined heat and power cycle steam turbine power plant was investigated in three different operation modes. The main technological benefit of cogeneration systems is their potential to enhance fuel efficiency in the production of electrical and thermal energy. In this context, the exergy concept is integrated with that of pinch analysis in process integration analysis to reduce fuel requirements (heat load) and to optimize the cogeneration cycle in steam turbine plants. Exergy analysis is carried out on all bottoming cycles to assess the exergy losses of the various components of the system. According to Bendig et al. (2012) [16], there is one holistic rule of exergy analysis aiming to minimize the area between the hot and cold composite curves of the integrated systems, including the energy recovery system. In the heat exchanger networking, the effect of temperature difference on the distance between the cold flow and heat flow is magnified by revealing that the greater the distance between the flows is, the greater the energy consumption and energy loss, and the lower the efficacy [17]. Hence, for the composite curve generation, an investigation of the fluctuation of the heating demand for a ∆*T_min_* of 10 °C was performed. Also, the GCC simplifies the identification of heat integration and energy recovery possibilities [5], and it is extremely valuable for a deeper understanding of the situation. Since utility prices are affected by temperature, higher-temperature hot utilities are often more expensive than lower-temperature hot utilities in terms of exergetic costs [18]. Cold utilities at lower temperatures, on the other hand, are more expensive than those closer to the ambient temperature.

The entire energy demand indicated by the composite curve might be provided at many levels, capable of computing the total energy objective, while it cannot specify the quantity of energy that should be given to the process at different temperatures, whereas the grand composite curve (GCC) defines the quantity of each, as discussed in Section 4.

### 3.4. Process Modification Solutions

Since the final purpose of this study is the detection of eventual process improvement solutions based on the results of the CPEA, some observations concerning possible modifications are discussed in this last subsection. The primary energy-saving computation was articulated by subsequent approaches for calculating electricity generation from the installed cogeneration plant in various scenarios. In particular, a cogeneration unit that is functioning with the greatest theoretically achievable heat recovery from itself is said to be in a full cogeneration mode (Grid: ON/ Factory: ON), and the process is considered combined heat and power (CHP). Sometimes, the overall efficiency of the cogeneration plant may fall below the threshold value (75–80%) [19]; the cogeneration plant is then said to be a non-CHP system. Therefore, the process modification principles must be implemented for maximum heat recovery in both CHP and non-CHP scenarios.

#### 3.4.1. Principles of Plus–Minus for Process Modification

In this principle, mostly the heat and material balance change, by shifting the position of composite curves with a subsequent impact on process energy targets. The +/− process modification decisions are made on the amount of electricity generated on site in order to sell any surplus from the three operational scenarios. The basic concept here is to modify the way energy is generated and how production is carried out in response to external factors, such as power costs related to exergetic losses [20]. Moreover, the design of an appropriate heat recovery network can help in meeting the minimum theoretical energy requirement and the reduction in exergy losses due to irreversibilities. However, by employing thermodynamic criteria based on pinch analysis, it is feasible to find modifications in the relevant process parameters which will reduce energy requirements that could be governed by the plus–minus principle.

Useful guidelines in order to carry out this task are, firstly, that any increase in hot stream duty above the pinch point and decrease in cold stream duty above the pinch point results in a reduced hot utility target. Secondly, any increase in hot stream duty above the pinch region and any decrease in cold stream duty above the pinch region results in a lower hot utility target. A lowered cold utility is also the outcome of a drop in hot stream duties below the pinch region and an increase in cold stream duties below the pinch area [21].

#### 3.4.2. Heat Pump Integration

Process utility systems rely heavily on some critical units such as heat engines and heat pumps. As Gundersen (2013) [6] stated, the heat pumps should be integrated over the pinch in such a way that it takes heat from the surplus zone below the pinch and transfers it to the deficit region above the pinch. Such an integration mechanism helps to reduce hot and cold fluid consumption. In general, heat transfer over a temperature difference results in exergy destruction owing to friction and material degradation. It is recommended that a correctly constructed heat integration network along with strategically located steam extraction (heat engine) stations can improve the system’s heat integration [22]. In addition, Tiwari et al. (2012) [23] pointed out that the temperature differential between the cold and hot streams is also greater at higher pinch points, resulting in larger irreversibilities. As a result, the optimal placement of heat pumps (heat engines) in a particular system might be in two distinct locations according to the best compromise between process heat demand minimization and maximum power generation. One of the most energy efficient combinations of process heat demand and the generation of power is heat integration. Since it is desirable to reduce the hot utility demand, the heat engine must be placed above the pinch temperature to reject heat into the process and the heat transferred to the heat sink. On the other hand, when the heat engine is placed below the pinch temperature, it brings energy from the process of an overall heat source. On the contrary, the integration of a heat engine across the pinch does not furnish any benefit.

The results of the cogeneration plant CPEA analysis and the subsequent detection of process improvements are therefore discussed in the following section.

## 4. Results and Discussion

In this section, the results obtained for the three alternative scenarios are discussed according to the pinch, exergy, and CPE analysis, based on the ProSimPlus^®^ simulation results. The outcome will be analyzed and commented upon in conformity with the final purpose of this study, i.e., the assessment of the efficiency for retrofitting and the opportunity to improve the system performance by applying reasonable modifications. However, before addressing the energy optimization problem, some simulation results need to be discussed in order to have a better understanding of the phenomenological behavior of the system. The obtained flowrate values for the most relevant process streams and the power generated for each turbine section are then reported in Table 1 according to the system functioning mode.

As already mentioned in the case study section, it can be noticed that the circulating flowrate is considerably affected by the amount of power that needs to be generated. However, the plant working at full capacity requires less steam than the exact proportional amount with respect to the other functioning modes. This aspect can already be interpreted as a symptom of higher efficiency, which will be later confirmed by the CPEA study. Moreover, although the total electricity production decreases in the case of a non-operational sugar factory (cf Scenario II), the power distribution between the three turbine sections is more homogeneous. In fact, since no steam should be sent to the factory, no constraints related to its pressure are applied at the second expansion.

Furthermore, it can be pointed out that the total electricity production at full capacity is higher than the declared 31.5 MW value. The first reason for overproduction is the need for producing the electricity that is consumed by pumps and other units within the CHP plant. In addition, the mismatch can be explained by the fact that the simulation results provide the ideal value of generated power without considering dispersions, which should be compensated by a corresponding production surplus. In fact, in the case that the grid connection is off (cf Scenario III), the 12 MW of total generated power is only slightly higher than the 11 MW served to the sugar factory.

With regard to the exergy and the thermal energy balances, exergy losses are provided in Section 4.2 while the heat capacity and enthalpic flows related to each stream are provided in Appendix A and discussed in the following section.

### 4.1. Targeting by Pinch

**Figure 5:**
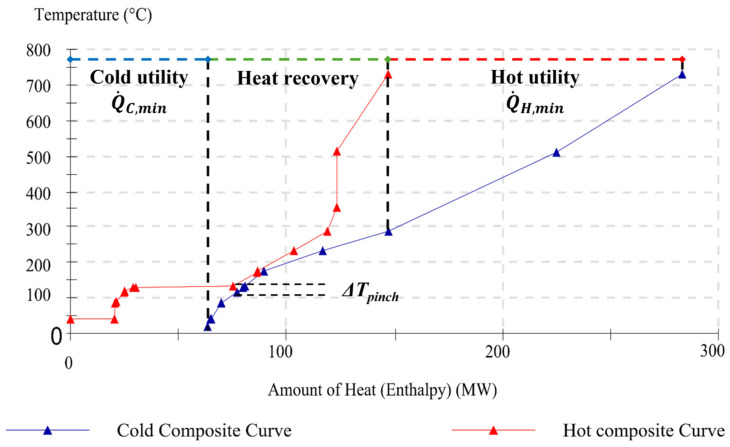
Hot and cold composite curves for Scenario I (Grid: ON and Factory: ON).

**Figure 6:**
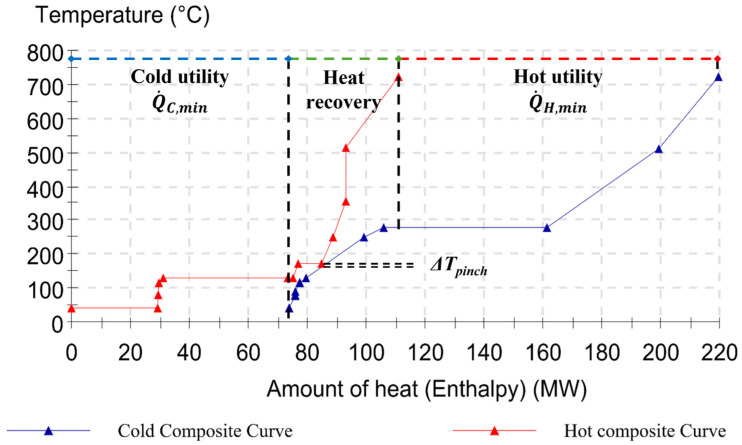
Hot and cold composite curves for Scenario II (Grid: ON and Factory: OFF).

**Figure 7:**
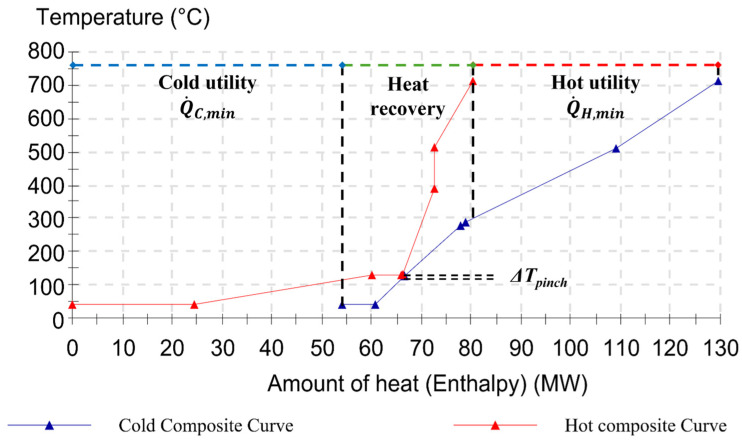
Hot and cold composite curves for Scenario III (Grid: OFF and Factory: ON).

As a first result, the composite curves for each scenario are built according to the temperature and enthalpy levels of the process streams, as reported in Figure 5, Figure 6 and Figure 7 (cf Appendix A for numerical values and stream properties). The specification of the minimum permissible temperature difference being equal to ∆*T_lm_* = 10 °C, which is an economic parameter for the trade-off between investment costs (heat exchangers) and running costs (energy), determines the targets for the heat recovery system. Based on this hypothesis, the minimal external heating (${\dot{Q}}_{H,\min}$) and minimum external cooling (${\dot{Q}}_{C,\min}$) requirements can be assessed either directly from the CCs’ graphics or by calculating the energy balances for each heat transfer section. These two values indeed are represented by the two parts where there is no curve overlapping, and they are equal to

- ${\dot{Q}}_{H,\min}$ = 135.76 MW and ${\dot{Q}}_{C,\min}$ = 63.28 MW with a pinch point at 185 °C for Scenario I (cf Figure 5);
- ${\dot{Q}}_{H,\min}$ = 108.73 MW and ${\dot{Q}}_{C,\min}$ = 79.7 MW with a pinch point at 171 °C for Scenario II (cf Figure 6);
- ${\dot{Q}}_{H,\min}$ = 108.73 MW and ${\dot{Q}}_{C,\min}$ = 49.10 MW with a pinch point at 131 °C For Scenario III (cf Figure 7).

In this instance, the satisfaction ratio in Scenario I achieved 18.24% and 12.20% for cold and hot fluids, respectively. Hence, with the assistance of the pinch analysis design and targeting skills, combined exergy and pinch analysis may provide an enhanced use of the exergy idea rather than pure thermal analysis, as accurately discussed in the next sections.

### 4.2. Exergy Analysis

Besides the pinch analysis, concerning the energy and temperature distribution of the process streams, the exergy analysis was carried out to better understand the unit-wise impact of equipment inefficiencies for each functioning mode. In particular, while the former was performed to optimize the heat transfer mechanism between cold and utilities for heat recovery, the latter aims at the identification of exergy destruction in the components of the cycle in order to identify the equipment that should be eventually improved.

As previously mentioned, the exergy analysis was performed based on the simulation results via the “Exergy balance” built-in tool of the ProSimPlus^®^ process simulator for each scenario. The obtained results are reported in Table 2 only for units with non-zero exergy destruction values.

As a first remark, it can be immediately noticed that the three scenarios exhibit analogous exergy destruction distribution over the different modules (with the exception of the inactive ones). In general, it can be observed that pumps have a low impact on exergy losses and that this value is mainly proportional to the circulating flowrate, as for the deaerator and the mixers in the first two scenarios. On the contrary, in Scenario III, the mixer inefficacy increases due to the relevant thermal difference between the streams to be mixed. In addition, it can be observed that there are some specific units which play a role of major impact in terms of exergy destruction. In particular, the heat exchanger HEX1-HP accounts for around 31% of total exergy destruction, followed by HEX2-LP. Since both these exchangers are dedicated to heat recovery, it can be concluded that the energy integration already present in the cycle is not optimal for the energy efficiency of the process. Furthermore, a considerable contribution can be pointed out for the turbine sections T-1, T-2, and T-3, which are responsible for 30%, 14%, and 8% of the plant’s exergy destruction, respectively. However, the irreversibility due to the turbines can be mitigated only by means of an equipment efficiency improvement, e.g., unit replacement.

The analysis coupling both the exergy and pinch results is therefore discussed in the next section to comment on additional aspects aimed at the improvement of the process layout.

### 4.3. Combined Exergy and Pinch Analysis

The general purpose of this approach is the estimation of the entire avoidable and unavoidable exergy losses for the global process and the specific process units, revealing potential improvements for heat recovery by means of an exchanger network (HEN).

As discussed in the previous sections, for this specific case study, Scenario I exhibits particular potential in terms of energy savings and integration. The impact of the ∆*T_lm_* affects the quantity of energy and exergy level in the hot and cold streams, particularly for steam turbine extraction steam, low-pressure steam (LP), intermediate pressure (IP), and high-pressure (HP) steam. In fact, this is the only aspect playing a critical role in the heat transfer and heat recovery capacity reduction, resulting in exergy losses. Thus, the heat duty and maximum recovery along with its integration satisfaction ratio from the extraction and condensate streams have been collected for the three operational scenarios.

Moreover, for the cogeneration plant, it can be observed that pinch analysis is able to minimize energy usage in electricity production by enhancing energy recovery. Before moving to the quantitative analysis of the outcome, it is worth noting that Scenario I already includes a heat recovery loop for the exchangers HEX1-HP and HEX2-LP, with energy savings corresponding to 23.7 MW and 3.06 MW, respectively.

However, based on the results concerning units’ irreversibility, it can be also noted that some unit operations do not need to be included in the process system integration, while it would be better to redesign or directly exclude them from the economic opportunities point of view. For instance, Mixer-1 and the deaerator in Scenario I; Mixer-1, Mixer-2, the condenser, and the deaerator in Scenario II; and finally, Mixer-1 in Scenario III must be either redesigned or excluded from the system to maximize the cogeneration system economic advantage over the equipment purchase cost.

Furthermore, the pressure (or saturated temperature) and the extraction mass flowrates were fixed at the optimal position of the turbine blades (at 9 and 2.6 bar) in order to utilize the minimum amount of fuel (heat duty) for the boiler feed water heating. Table 3 and Table 4 show the extraction quantities, saturation temperatures, and the quantity of heat (heat duty) consumed by the cogeneration plant in each scenario.

In case the cost of energy was higher than the selected value, the targeted investment would overcome the maximum threshold even in case of 32% integration ratio (Table 4, Scenario I) at a pinch point temperature of 185 °C. In this situation, Scenario I is the most feasible integration configuration, with a maximum energy recovery equal to 83.44 MW and integration potential indicators #1 and #2 up to 45.61% and 29.54%, respectively.

The possibility of heat pump integration is then discussed in the next section.

### 4.4. Heat Pump Placement

For both the heat supply of the process and power generation, the GCC can help locate the heat pump position in a process system. The most energy efficient combination for this purpose is achieved by integrating heat pumps to allow waste heat to be used for process heating. However, before exploiting the waste heat (represented in this research by extracts and condensate recycling), economic drawbacks must be considered. The economics of heat pump placement are indeed determined by the balance of process heat savings versus the electricity consumption for heat pumping. To make the heat pump alternative cost-effective, a high process heat duty and a small temperature difference across the heat pump cycle are required.

**Figure 8:**
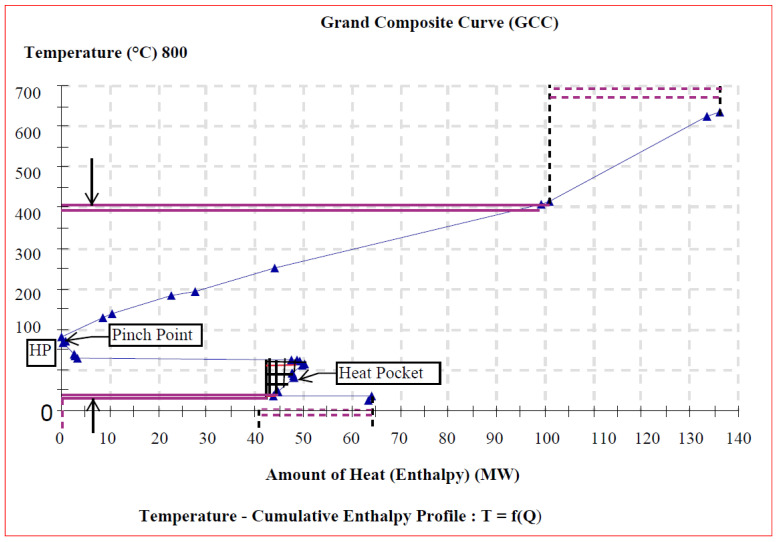
GCC with heat pocket for heat pump integration (Scenario I).

**Figure 9:**
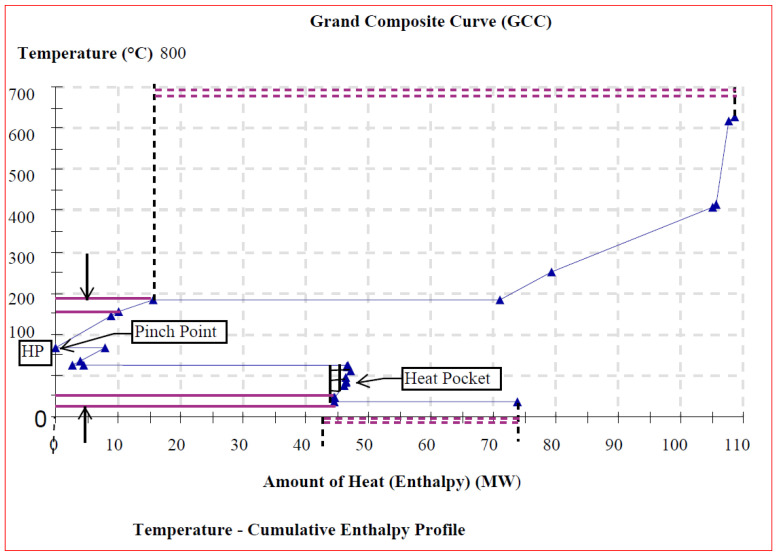
GCC with heat pocket for heat pump integration (Scenario II).

**Figure 10:**
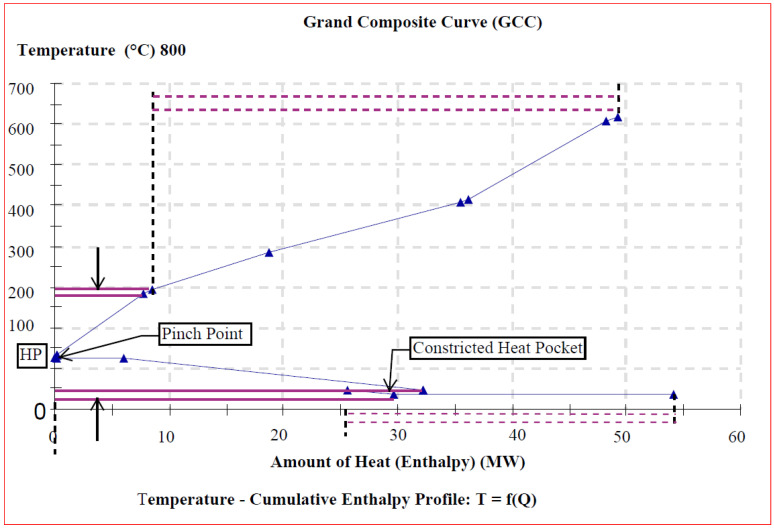
GCC with heat pocket for heat pump integration (Scenario III).

Figure 8, Figure 9 and Figure 10 depict the background process’s grand composite curves (GCCs) for the three scenarios, respectively. They were used to determine whether heat pumps could enhance the cogeneration system economic benefit via energy saving. This graphic contains the same basic data of interest as the composite curves (CCs) (i.e., the position of the pinch and the minimal external heating and cooling), but it additionally conceals information about process-to-process heat transfer. For each of the obtained GCC graphics, a net heat surplus can be derived by transferring the surplus heat from one interval to another with a heat deficit at lower temperature, by forming a special feature heat pocket below the pinch region.

In this study then, the GCCs not only display how much external heating and cooling is necessary, but they also illustrate at what temperatures such external heating and cooling are needed. In Scenarios II and III, above and below the pinch, the GCC exhibits an area of little temperature change and substantial enthalpy change. However, in Scenario I, the area of high temperature change does not correspond to a significant enthalpy change compared with the other two operational scenarios. In Figure 8, Figure 9 and Figure 10, the pointed “nose” at the pinch suggests that a heat pump may be placed for reasonably considerable savings in heating (${\dot{Q}}_{H,\min}$) and cooling (${\dot{Q}}_{C,\min}$) demand throughout the modest temperature shift. As a result, the energy savings will be significant for a small power consumption, leading to high performance. Therefore, the integration of a heat pump in Scenarios II and III is quite beneficial in terms of heat recovery. However, in Scenario I, the heat pump alternative appears to be uneconomical since the temperature differential across the heat pump is fairly wide, resulting in a high power consumption of the additional unit.

## 5. Conclusions

In this research, the implementation of the exergy concept aligned with pinch-based methodologies for analyzing the optimal integration of energy conversion systems in steam turbine cogeneration plants was investigated. The methodology proved to be effective for the desired purpose and, based on the obtained outcome, some important conclusions of general validity can be drawn:

- The application of the CPEA method successfully allows the detection of steam usage reduction opportunities and effective waste heat recycling requirements by means of dedicated heat integration;
- For each specific operating mode, the maximum energy recovery, and thus steam consumption, boundary can be quantitatively estimated. In particular, for the Wonji-Shoa cogeneration plant, up to 83.44 MW, 36.88 MW, and 26.15 MW can be recovered for Grid/Factory scenarios ON/ON, ON/OFF, and OFF/ON, respectively. These values represent a highly significant increase in cogeneration system efficiency and potential electricity surplus from the already installed condensing and extracting turbines;
- In addition, the CPEA method allows the quantification of the energy-saving potential indicators in case of a plant revamping decision. In fact, although Scenarios II and III already include waste heat recycling and reuse, the analysis highlighted further heat savings in the case a better energy integration is implemented. For instance, Scenario I exhibits integration potential indicators #1 and #2 equal to 45.61% and 26.54%, respectively;
- The highest exergy destruction cannot always be correlated with the highest energy recovery potential. As for this specific case study, even if Scenario III exhibits the highest irreversibility, the operating mode with the largest potential for irreversibility reduction is Scenario II;
- In terms of methodology, each tool can be correlated to a specific purpose. For pinch analysis, the composite curves (CCs) are used to calculate the process’s lowest energy and exergy demand. First, part of the exergy requirement is calculated by accounting for an exergy loss caused by the differential temperature (∆*T_ml_*). Then, the remaining part is given by the sum of three contributions: the exergy created as an energy surplus between the pinch point and the ambient temperature, the exergy required above the pinch point, and the exergy required for minimal cooling and heating. In this case, the GCC diagrams are used to calculate the process’s net cumulative heat surplus and heat deficit, serving as a useful interface between the process and the utility sections.

Based on these observations, the CPEA approach is worth further studies for more detailed and complex applications. In particular, it would be interesting to assess how the results in terms of optimal energy savings and exergy recovery could be exploited as a decisional tool for the selection of process and utility system operating modes and related optimal scheduling. This analysis, coupled with economic and environmental indicators, could exhibit great potential in terms of simultaneous profitability and sustainability optimization.

## Figures and Tables

**Table 1:** Flow summary and generated power for the CHP section.

Stream	Scenario I	Scenario II	Scenario III
Circulating flowrate [t/h]	165	130	59
HP steam extraction [t/h]	20	14	0
LP steam extraction [t/h]	115	72	43
T-1 steam inlet [t/h]	165	130	59
T-2 steam inlet [t/h]	145	116	59
T-3 steam inlet [t/h]	30	44	16
T-1 power [MW]	22.53	16.9	6.4
T-2 power [MW]	11.98	9.58	3.8
T-3 power [MW]	4.83	7.07	2.1

**Table 2:** Exergy loss values in process equipment [MW].

Unit	Scenario I	Scenario II	Scenario III
Deaerator	0.5309	0.0550	/
Pump-1	0.0029	0.0039	0.0049
Pump-2	0.1920	0.1217	0.0573
T-1	3.0204	2.2313	1.6772
T-2	1.4339	2.2313	1.0440
T-3	0.8384	1.2296	0.9454
Mixer-1	0.5669	0.0269	4.5162
Mixer-2	0.0454	0.0075	/
HEX1-HP	3.1515	2.2257	0.8308
HEX2-LP	0.3249	0.2924	0.5585

**Table 3:** Maximum heat energy recovery for the hot and cold sides.

Scenario	Fluid	Heat Duty [MW]			Satis. Ratio [%]
		Minimum	Actual	Maximum	
I	Cold	63.28	119.96	146.71	18.24
	Hot	135.76	192.44	219.19	12.21
					
II	Cold	79.66	/	118.57	/
	Hot	108.73	/	147.64	/
					
III	Cold	53.97	/	80.12	/
	Hot	49.10	/	75.25	/

**Table 4:** Maximum heat energy recovery and integration potential.

Property	Scenario I	Scenario II	Scenario III
Max energy recovery [MW]	83.44	36.88	26.15
Pinch temperature [°C]	185	171	131
Actual integration ratio [%]	32.07	/	/
IPI ^1^ # 1 [%]	45.6	/	/
IPI ^1^ # 2 [%]	29.5	/	/

^1^ IPI stands for integration potential indicator.

## Data Availability

Data are contained within the article.

## Glossary

Symbol	Definition	Unit
*B_ch_*	Chemical exergy	MW
*B*_(*D*,*pump*)_	Exergy destruction rate of pump	MW
*B_fuel_*	Fuel exergy (heat duty)	MW
*B_heat_*	Exergy of heat stream	MW
*B*_(*İ*,*HE*)_	Exergy of heat exchanger	MW
*B_material_*	Exergy of material stream	MW
*B_ph_*	Physical exergy	MW
*B*_(*P*,*k*)_	Component’s exergy	MW
*B_system_*	Exergy of the system	MW
*B^T^*	Physical exergy due to temperature change	MW
*B_work_*	Work rate	MW
*b_i_*	Specific exergy	MW
CC	Composite curve	acronym
CCC	Cold composite curve	acronym
*C_p_*	Specific heat capacity	(kJ/kg. K)
CPEA	Combined pinch and exergy analysis	acronym
${\dot{E}}_{\mathrm{in}}$	The rate of energy input to the plant	MW
GCC	Grand composite curve	acronym
*h*	Specific enthalpy at a temperature T	(KJ/kg)
*h*_0_	Specific enthalpy at a temperature T_0	(KJ/kg)
HCC	Hot composite curve	acronym
HEN	Heat exchanger network	acronym
$\dot{Q}$	Exergy destruction rate (irreversibility)	MW
*ṁ*	Mass flow rate	(t/h)
$\dot{Q}$	Heating power	MW
*S*	Entropy	(kJ/kg. K)
*S*_0_	Entropy at a dead state temperature	(kJ/kg. K)
*T*_0_	Temperature at dead state	°C
*T_i_* (*i =* 1, 2, 3)	Turbine units (1, 2, and 3)	-
${\dot{W}}_{\mathrm{cv}}$	Rate of work performed in control volume	MW
${\dot{W}}_{\mathrm{net}}$	Net power output	MW
${\dot{W}}_{\mathrm{out}}$	The actual generated expansion work	MW
${\dot{W}}_{in}$	The actual power consumed in the pump	MW
*X_k_*	Mass fraction of component k-th	kg/kg
*η_cogen_*	Energy efficiency of the cogeneration facility	-
*η_cycle_*	Overall exergetic efficiency of the cycle	-
*η_pump_*	Pump’s exergetic efficiency	-
*ΔT_lm_*	Logarithmic mean temperature difference	°C

## Appendix A

This appendix reports thermal data for each heat exchanger network stream according to the cogeneration plant functioning mode.

**Table A1:** Stream result data for the HEN (Scenario I, cf Figure 2).

#	Type	State	*ṁ·C_p_* [MW/°C]	*T_in_* [°C]	*T_out_* [°C]	$\dot{Q}$ [MW]
31	Cold	V	0.0386	134.105	138.053	0.1526
27	Cold	L	0.0419	46.156	121.091	3.0620
20	Cold	L	39.0877	46.065	46.075	0.3909
1	Cold	L	0.0628	25.000	90.336	4.1029
3	Cold	L	0.2081	91.803	291.477	41.5614
10	Cold	V	0.0863	237.251	511.982	23.6963
5	Cold	L	0.2656	180.000	730.537	146.2280
4	Hot	L	0.2181	291.477	180.000	24.3143
13	Hot	V	0.2250	175.949	138.053	8.5279
23	Hot	V	0.3058	134.105	124.091	3.0620
19	Hot	LV	1983.750	46.085	46.075	19.8375
24	Hot	L	0.0058	124.091	46.075	0.4557
28	Hot	L	0.0411	121.091	95.000	1.0726
30	Hot	V	11.3234	138.053	134.005	45.8372
14	Hot	L	0.0895	134.005	90.336	3.9057
29	Hot	L	0.04090	95.000	90.336	0.1908
25	Hot	LV	45.6022	46.085	46.075	0.4560
35	Hot	V	0.0091	237.251	175.949	0.5561
6	Hot	V	0.1099	730.537	514.982	23.6963
15	Hot	V	0.0659	358.743	134.005	14.8014

**Table A2:** Stream result data for the HEN (Scenario II, cf Figure 3).

#	Type	State	*ṁ·C_p_* [MW/°C]	*T_in_* [°C]	*T_out_* [°C]	$\dot{Q}$ [MW]
27	Cold	L	0.0546	46.15	82.28	1.9738
20	Cold	L	12.8248	46.06	46.08	0.1283
29	Cold	L	0.0552	95.00	119.99	1.3791
3	Cold	L	0.1646	121.36	281.77	26.4079
4	Cold	L	0.2209	281.77	723.08	97.4858
10	Cold	V	0.0689	253.27	511.98	17.8217
13	Hot	V	788.9600	175.96	175.95	7.8896
22	Hot	V	0.0405	134.01	85.28	1.9738
5	Hot	V	640.2730	134.02	134.00	6.4027
14	Hot	L	0.0168	175.95	134.00	0.7056
1	Hot	L	0.0035	85.28	46.08	0.1368
30	Hot	LV	4215.2100	134.02	134.00	42.1521
21	Hot	L	0.0983	134.00	119.99	1.3773
25	Hot	LV	13.6991	46.09	46.08	0.1370
12	Hot	V	0.0090	253.27	175.95	0.6931
19	Hot	LV	2909.5000	46.09	46.08	29.0950
6	Hot	V	0.0857	723.08	514.99	17.8226
15	Hot	V	0.0412	358.74	134.00	9.2667

**Table A3:** Stream result data for the HEN (Scenario III, cf Figure 4).

#	Type	State	*ṁ·C_p_* [MW/°C]	*T_in_* [°C]	*T_out_* [°C]	$\dot{Q}$ [MW]
27	Cold	L	0.0690	46.16	131.01	5.8536
3	Cold	L	0.0751	132.39	281.77	11.2178
5	Cold	L	0.1017	281.77	713.18	43.8573
20	Cold	L	661.7250	46.07	46.08	6.6173
9	Cold	V	0.0350	291.83	511.99	7.7020
2	Hot	V	585.3600	134.02	134.01	5.8536
24	Hot	LV	0.2775	134.01	46.08	24.3982
25	Hot	LV	2440.1300	46.09	46.08	24.4013
6	Hot	V	0.0389	713.18	514.99	7.7020
19	Hot	V	0.1267	135.97	46.08	11.3895
15	Hot	V	0.0246	392.69	134.01	6.3742

## References

[1] Di Pretoro A., Fedeli M., Ciranna F., Joulia X., Montastruc L., Manenti F. (2022). Flexibility and environmental assessment of process-intensified design solutions: A DWC case study. Comput. Chem. Eng..

[2] Di Pretoro A., Manenti F. (2020). Pinch Technology. Non-Conventional Unit Operations. SpringerBriefs in Applied Sciences and Technology.

[3] Zhao Y., Zhang Y., Cui Y., Duan Y., Huang Y., Wei G., Mohamed U., Shi L., Yi Q., Nimmo W. (2022). Pinch combined with exergy analysis for heat exchange network and techno-economic evaluation of coal chemical looping combustion power plant with CO_2_ capture. Energy.

[4] Linnhoff B., Townsend D.W., Boland D., Hewitt G.F., Thomas B.E.A., Guy A.R., Marsland R.H. (1982). User Guide on Process Integration for the Efficient Use of Energy.

[5] Pina E.A., Palacios-Bereche R., Chavez-Rodriguez M.F., Ensinas A.V., Modesto M., Nebra S.A. (2017). Reduction of process steam demand and water-usage through heat integration in sugar and ethanol production from sugarcane—Evaluation of different plant configurations. Energy.

[6] Gundersen T., Klemes J. (2013). 4—Heat Integration—Targets and Heat Exchanger Network Design. Woodhead Publishing Series in Energy, Handbook of Process Integration.

[7] (2013). Wonji-Shoa factory, Operational manual of Wonji-Shoa sugar factory.

[8] Sharew S., Montastruc L., Yimam A., Negny S., Ferrasse J.-H., Turkay M., Gani R. (2021). Optimal efficiency of biomass conversion from bio-based byproducts to biofuel production in the ethiopian sugar industry: A case study in Wonji-Shoa sugar factory, Ethiopia. 31st European Symposium on Computer Aided Process Engineering of Computer Aided Chemical Engineering.

[9] Chao Z., Yan W. (2006). Exergy cost analysis of a coal-fired power plant based on structural theory of thermoeconomics. Energy Convers. Manag..

[10] Ensinas A.V., Modesto M., Nebra S.A., Serra L. (2009). Reduction of irreversibility generation in sugar and ethanol production from sugarcane. Energy.

[11] Kanoglu M., Dincer I. (2008). Performance assessment of cogeneration plants. Energy Convers. Manag..

[12] Kotas T.J. (1985). The Exergy Method of Thermal Plant Analysis.

[13] Sanjay Y., Singh O., Prasad B.N. (2007). Energy and exergy analysis of steam cooled reheat gas–steam combined cycle. Appl. Therm. Eng..

[14] Manassaldi J., Mussati S., Scenna N. (2010). Optimal synthesis and design of Heat Recovery Steam Generation (HRSG) via mathematical programming. Energy.

[15] Chen X., Chang C., Wang Y., Feng X. (2016). An energy hub approach for multiple-plants heat integration. Chem. Eng. Trans..

[16] Bendig M., Marechal F., Favrat D. (2012). Defining the Potential of Usable Waste Heat in Industrial Processes with the Help of Pinch and Exergy Analysis. Chem. Eng. Trans..

[17] Rudiyanto B., Raga T., Prasetyo T., Eko Rahmanto D., Nuruddin M., Pambudi N., Wibowo K. (2020). Analysis Heat Exchanger Network Steam Power Plant in Using Pinch (case study in PT POMI Unit 3 power Plant Paiton). Int. J. Heat Technol..

[18] Wan Alwi S.R., Zainuddin A.M. (2016). Simultaneous energy targeting, placement of utilities with flue gas, and design of heat recovery networks. Appl. Energy.

[19] Gambini M., Vellini M. (2014). High Efficiency Cogeneration: Performance Assessment of Industrial Cogeneration Power Plants. Energy Procedia.

[20] Pablos C., Merino A., Acebes L.F. (2019). Modeling On-Site Combined Heat and Power Systems Coupled to Main Process Operation. Processes.

[21] Lythcke-Jørgensen C.E., Haglind F., Clausen L.R. (2014). Exergy analysis of a combined heat and power plant with integrated lignocellulosic ethanol production. Energy Convers. Manag..

[22] Hamssin F. (2017). Analysis of Pinch and Approach point of Heat Recovery Steam Generator (HRSG) of cogeneration plant.

[23] Tiwari A.K., Hasan M.M., Islam M. (2012). Effect of Operating Parameters on the Performance of Combined Cycle Power Plant. Sci. Rep..

